# Posttraumatic Stress Disorder Symptoms and Alcohol Use Among Korean Firefighters: The Roles of Coping Motives for Drinking and Family Support

**DOI:** 10.3390/healthcare14040421

**Published:** 2026-02-07

**Authors:** Nayoon Lee, Kyoung-eun Lee

**Affiliations:** 1 College of Nursing, Dong-A University, Busan 49201, Republic of Korea; 095750@dau.ac.kr; 2 Department of Nursing Science, SunMoon University, Asan-si 31460, Republic of Korea

**Keywords:** firefighters, posttraumatic stress disorder symptoms, alcohol use, coping motives, family support, sleep quality

## Abstract

**Highlights:**

**What are the main findings?**

- Post-traumatic stress symptom severity was positively associated with alcohol use among Korean firefighters, with coping motives for drinking showing a significant mediating effect.
- Family support significantly moderated the relationship between post-traumatic stress symptoms and alcohol use, whereas sleep quality did not show a significant mediating role.

**What are the implications of the main findings?**

- Interventions focusing on coping-related drinking motives may help reduce alcohol misuse among firefighters with elevated post-traumatic stress symptoms.
- Strengthening family support should be considered a key component of mental health and alcohol prevention programs for firefighters.

**Abstract:**

**Background/Objectives**: This study aimed to analyze the effects of coping motives for drinking, sleep quality, and family support on the relationship between PTSD symptoms and alcohol use among Korean firefighters. **Methods**: Data from 600 firefighters in a specific region of Korea were collected. Descriptive statistics, correlation coefficients, and bootstrapping were used to analyze the data and PROCESS macro to verify the mediating effect of coping motives for drinking and sleep quality, as well as the moderating effect of family support. PTSD symptoms were measured using the PCL-5 (range = 0–80; probable PTSD ≥ 33; total score: 33.2 ± 15.2) and alcohol use was measured using the AUDIT-K (range = 0–40; risky drinking ≥ 8; total score: 6.8 ± 6.2). **Results**: PTSD symptom severity was significantly associated with alcohol use, and coping motives for drinking showed a significant indirect association in the relationship between PTSD symptoms and alcohol use. However, sleep quality did not show a significant indirect association, and family support significantly moderated the association between PTSD symptom severity and alcohol use. **Conclusions**: Findings underscore the importance of addressing coping-motivated drinking and strengthening family support when managing alcohol problems associated with PTSD symptoms in firefighters. Education on adaptive stress-coping alternatives and family-centered support may help prevent alcohol misuse.

## 1. Introduction

Firefighters experience significantly higher physiological and psychological stress levels because of the nature of their profession, which requires quick responses and coping skills. Consequently, relatively higher levels of depression and anxiety have been reported among firefighters compared to other professionals [1]. Because of their repeated exposure to traumatic situations that most individuals may never experience, firefighters are considered a highly vulnerable group at risk of posttraumatic stress disorder symptoms (PTSD symptoms) and other mental health problems [2]. PTSD symptoms can develop after experiencing extremely life-threatening or terrifying events [3]. Previous studies have reported that firefighters commonly use alcohol to deal with exposure to trauma and stress at work [4,5]. Among firefighters in the United States (U.S.), 50% were reported as highly prone to experiencing heavy drinking and alcohol use problems [5,6]. The proportion of Korean firefighters with problematic alcohol consumption was reported as 26.4% [3]. Moreover, more active interventions are required for individuals with comorbid PTSD and alcohol misuse, as this association is linked with increased mental health risks, including suicidal behaviors [7].

Other variables associated with PTSD symptoms and alcohol misuse among firefighters include coping motives for drinking and sleep quality. Individuals with coping motives for drinking choose alcohol consumption to cope with stress or negative affective states. This variable can directly explain drinking-related behaviors [8]. Firefighters tend to drink alcohol excessively to cope with stress and negative events [9], whereas those with PTSD symptoms report drinking alcohol to cope with PTSD-related symptoms [10]. Moreover, as firefighting is a demanding occupation with high stress levels, compounded by shift work, the prevalence of sleep disturbance among firefighters is significantly high. Owing to the seriousness of these problems, several studies have examined sleep problems among firefighters [11,12,13].

However, while poor sleep is often discussed as a consequence of alcohol use, several studies suggest that it may also precede and contribute to increased drinking by impairing emotional regulation and increasing stress vulnerability, particularly in high-risk occupations such as firefighting [14]. In addition, Sleep quality may serve as a mediator in the pathway from PTSD symptoms to problematic alcohol use, as prior research has demonstrated a strong bidirectional association between sleep disturbances and substance use [12].

Family support has been identified as a protective factor that enhances health-related quality of life and reduces the prevalence of post-traumatic stress disorder (PTSD) among firefighters [15,16], and it is positively associated with sleep quality [17,18]. It also fosters healthier coping by discouraging maladaptive strategies such as alcohol use in stressful situations [19]. According to the social support deterioration model [20], social support may function as a mediator. In addition, strong family support has been shown to buffer the adverse effects of PTSD symptoms on maladaptive behaviors such as alcohol misuse, suggesting that social support may also operate as a moderator [21]. However, few studies have empirically examined how family support moderates the association between PTSD symptoms and alcohol use, specifically among firefighters.

**Figure 1:**
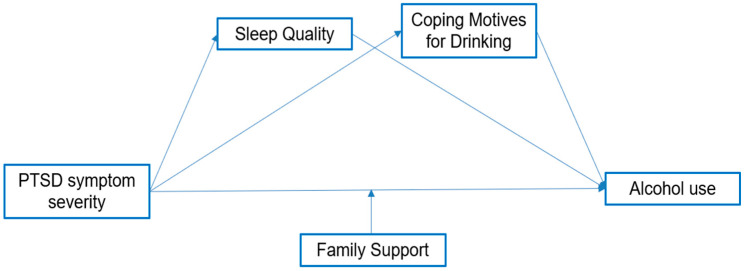
The conceptual model.

Understanding the mechanisms through which PTSD symptoms lead to problematic alcohol use is therefore crucial for designing tailored nursing and mental health interventions that address both individual and family contexts. To this end, the present study conceptualized a hypothetical model (Figure 1) incorporating PTSD symptoms, coping motives for drinking, sleep quality, and family support. In this framework, sleep quality and coping motives for drinking were tested as mediators, and family support as a moderator of the relationship between PTSD symptoms and alcohol use. PTSD symptoms were operationalized as PTSD symptom severity, measured using the PTSD Checklist for DSM-5 (PCL-5).

Specifically, the present study aims to:

(1) Determine the correlations between alcohol use and PTSD symptom severity, coping motives for drinking, sleep quality, and family support.
(2) Validate the mediating effect of sleep quality and coping motives for drinking and the moderating effect of family support on the relationship between PTSD symptom severity and alcohol use among firefighters.

## 2. Materials and Methods

### 2.1. Study Subjects

Career firefighters working in city B, Korea, were recruited for this study. The participants understood the study’s purpose and provided consent to participate. Eligibility required any lifetime alcohol use; accordingly, lifetime abstainers were excluded because coping motives for drinking can only be assessed among individuals with alcohol consumption experience. As a result, the target population of this study was firefighters with a history of alcohol use rather than the entire firefighter workforce.

At the planning stage, we anticipated a multivariable regression model with a relatively large number of predictors and interaction terms and therefore used a conservative rule of thumb of 10–15 participants per estimated regression coefficient, as commonly recommended for multivariate regression analyses [22]. Under this conservative scenario, the minimum required sample size was approximately 330 participants. Because standard priori power analysis tools do not readily accommodate the full complexity of the conditional process model ultimately estimated in this study, which included direct effects, mediators, a moderator, and an interaction term, this calculation should be interpreted as a pragmatic planning heuristic rather than a formal power analysis for mediation and moderation effects [23].

In the final model, the most complex regression equation (alcohol use as the outcome) comprised seven predictors, including the focal variables, one interaction term, and two covariates (age and sex). Applying the same rule of 10–15 participants per coefficient to this equation yields a minimum requirement of 70–105 participants, and our final sample of 600 firefighters substantially exceeds this benchmark, providing a favorable ratio of observations to parameters for estimating individual-level associations and interaction effects. Participants were recruited from a single metropolitan fire department via a 72 h intranet posting, and all analyses were planned at the individual level. Although we did not perform a full a priori power analysis for the conditional process model, the achieved sample size of 600 firefighters substantially exceeds the minimum sample size implied by our planning heuristic and provides ample precision for estimating the paths in the conditional process model [22].

### 2.2. Study Instruments

#### 2.2.1. PTSD Symptom Severity

PTSD symptom severity was measured using the PTSD checklist-5 (PCL-5), a self-report measure developed by Weathers et al. [24]. The PCL-5 is an appropriate test for screening individuals for PTSD symptom severity, diagnosing PTSD, and monitoring changes in PTSD symptoms. The PCL-5 is a 20-item questionnaire. A PTSD diagnosis requires at least one B item (items 1–5), one C item (items 6–7), two D items (items 8–14), and two E items (items 15–20). The total symptom severity score was obtained by summing the scores for each of the 20 items. Each item was scored using a 5-point Likert scale ranging from 0 to 4, with a total score ranging from 0 to 80. A total score of 33 or higher is commonly used as the cutoff for probable PTSD [25]. Cronbach’s alpha was 0.94 in a previous study investigating the reliability and validity of the Korean version of PCL-5 [25], and Cronbach’s alpha was 0.97 in the present study.

#### 2.2.2. Sleep Quality

The Pittsburgh Sleep Quality Index (PSQI) was developed by Buysse et al. [26]. The Korean version of the PSQI (PSQI-K) was used in this study. The reliability and validity of the PSQI-K in assessing sleep quality had been tested by Sohn et al. [27]. The PSQI-K has 19 items grouped into seven subcategories, including subjective sleep quality, sleep latency, sleep duration, sleep efficiency, sleep disturbances, use of sleeping medication, and daytime dysfunction. Each item was scored on a 4-point Likert scale ranging from 0 to 3, and the total score ranged from 0 to 21, with higher scores indicating poorer sleep quality. The Cronbach’s alpha for the PSQI-K was 0.84, and that for the current study was 0.79.

#### 2.2.3. Alcohol Use

The Alcohol Use Disorders Identification Test (AUDIT), developed by the WHO, was used to screen for unhealthy alcohol use. We used the Korean version of AUDIT (AUDIT-K), validated by Lee et al. [28], showing high reliability and validity, to screen for alcohol use. The tool has 10 items grouped into three subcategories: hazardous drinking (three items), alcohol dependence (three items), and drinking-related problems (four items). Each item was scored using a 5-point Likert scale ranging from 0 to 4, and the total score ranged from 0 to 40, with higher scores indicating a greater likelihood of drinking. A total score of 8 or higher is the WHO-recommended cutoff for hazardous drinking, and this criterion is also widely used in Korea [28]. Cronbach’s alpha for the Korean version was 0.86, and that for this study was 0.87. In this study, the AUDIT-K was administered only to firefighters with a history of alcohol use, consistent with our eligibility criteria, and the findings should therefore be interpreted in the context of alcohol-using firefighters

#### 2.2.4. Coping Motives for Drinking

In this study, coping motives for drinking were assessed using a tool developed by Shin and Han [29], which is based on the drinking motives framework proposed by Cox and Klinger. The instrument comprises 16 items divided into four subcategories—enhancement, coping, social, and conformity motives—with each subcategory comprising four items. Among these, the present study only used the coping motives subcategory, as it is most directly related to stress-related drinking behaviors that are crucial in the hypothesized pathway from PTSD symptoms to alcohol use. Each item is rated on a 5-point Likert scale ranging from 1 (not at all true) to 5 (very true), with a total score ranging from 4 to 20. Higher scores indicate stronger drinking motives in the respective subcategories. Cronbach’s alpha for the coping motives subscale was 0.88 in the original study and 0.95 in the present study.

#### 2.2.5. Family Support

Family support was measured using the Social Support Scale developed by Park [30] and revised by Yoo [31]. We did not modify any items, response options, wording, or ordering. Instead, we implemented a referent-specific instruction asking participants to respond with reference to their family members only, thereby capturing perceived family support while preserving the instrument’s original content domains. This family-referent administration has precedent in Korea using the same scale [32] and was previously reviewed and approved in the first author’s doctoral dissertation [15]. This instrument comprised 25 items that assessed four types of support: emotional support (seven items), evaluative support (six items), informational support (six items), and instrumental support (six items). Each item was rated on a 5-point Likert scale ranging from 1 (strongly disagree) to 5 (strongly agree), with total scores ranging from 25 to 125. Higher scores indicate greater perceived family support. In the present study, Cronbach’s alpha was 0.85.

### 2.3. Data Collection and Ethical Considerations

This study was approved by the Institutional Review Board of the Catholic University of Pusan (IRB No. CUPIRB-2021-038-02) and conducted in accordance with the Declaration of Helsinki. Data collection was carried out from 1 to 3 September 2021.

To recruit participants, an online survey link was posted on the internal bulletin board of the City B fire department, which was accessible only through the organization’s intranet-based groupware system. An online survey approach was adopted in consideration of the study period, which coincided with strict COVID-19 social distancing policies in Korea, as well as the need to minimize in-person contact among firefighters due to operational and infection-control considerations. Upon accessing the link, participants were provided with detailed study information, including its purpose, data collection procedures, voluntary participation and withdrawal rights, confidentiality and anonymity, data retention and disposal, incentives, and the scope of personal information to be collected (e.g., phone number). The survey proceeded only if participants selected “Yes” to the consent question. All items were mandatory, the survey required approximately 15 min to complete, and a small gift was provided in appreciation. Access to the online survey was restricted to authorized firefighters via the intranet-based groupware system, and duplicate submissions were prevented by limiting responses to a single submission per Google account. All survey items were mandatory, resulting in no missing data among completed responses.

A total of 600 firefighters completed the survey, with no missing data or invalid responses. Although individual views, declines, or exclusions were not recorded, the intranet bulletin likely ensured broad visibility during the 72 h period, as firefighters routinely use the system for official communications and document processing.

Workforce counts for City B were obtained from the National Fire Agency statistical yearbook (2021) [33] (3631 active firefighters; 295 women, 8.1%). Because individual invitations, views, and declines were not tracked, a conventional response rate cannot be computed; instead, we report a completion proportion of 16.5% (600/3631).

### 2.4. Data Analysis Methods

The data collected were analyzed using IBM SPSS Statistics 24.0 (IBM, New York, NY, USA).

First, the participants’ demographics and work-related variables were analyzed using descriptive statistics. Second, descriptive analysis and Pearson’s correlation coefficients were used to determine the correlations between PTSD symptom severity, alcohol misuse, coping motives for drinking, family support, and sleep quality. Finally, the Hayes PROCESS Macro (PROCESS v3.3 by Hayes, Columbus, OH, USA) and the bootstrapping method were used to analyze the mediating effect of coping motives for drinking and sleep quality and the moderating effect of family support on the relationship between PTSD symptom severity and alcohol misuse [34]. Bias-corrected bootstrapping (10,000 samples, 95% confidence intervals) was applied, as it overcomes the limitations of the Sobel test, which assumes normality.

Given the cross-sectional design, all effects are interpreted as associations rather than causal relations, and indirect effects are considered statistical mediation, consistent with methodological cautions noted by Maxwell and Cole (2007) [35].

Also, assumption checks showed that all VIFs ranged from 1.13 to 1.71, indicating no multicollinearity. Although the Shapiro–Wilk test was significant (*p* < 0.001), which is common in large samples, visual inspection of residuals indicated only minor, acceptable deviations from normality. Generative artificial intelligence was not used in the study design, data collection, analysis, or interpretation of the data.

## 3. Results

### 3.1. General and Work-Related Characteristics

Among the 600 participants, the majority were male (87.83%), and most firefighters were in their 30s (43.83%) and 40s (26.00%). With respect to rank, firefighters (29.17%) and fire sergeants (25.50%) constituted the largest proportions of the sample, indicating a concentration in middle-ranking positions. Regarding primary job responsibilities, extinguishing fires (27.67%) and emergency medical services (26.83%) were the most common roles, followed by administrative duties (22.18%) and vehicle operation (15.33%).

In terms of work experience, over one-third of participants had less than five years of working experience (36.00%), while a substantial proportion reported long-term service of 20 years or more (18.16%). Firefighters also reported a high workload, with 36.00% responding to 11 or more emergency calls and 29.83% reporting 11 or more night shifts, indicating frequent nighttime duty. Detailed demographic and work-related characteristics are presented in Table 1.

These characteristics are presented to describe and contextualize the study sample. Subgroup comparisons were not conducted, as the primary focus of the analysis was on individual-level associations rather than between-group differences. According to the National Fire Agency Statistical Yearbook (2021) [33], the demographic composition of the study sample was generally comparable to that of the City B firefighter workforce, although differences were observed in gender and rank distributions (see Supplementary Table S1).

### 3.2. Characteristics and Correlations of PTSD Symptom Severity, Alcohol Use, Coping Motives for Drinking, Family Support, and Sleep Quality

Table 2 presents the descriptive statistics of the variables. The mean score of PTSD symptom severity, alcohol use, coping motives for drinking, family support, and sleep quality was 1.66 ± 0.76, 0.68 ± 0.62, 2.31 ± 0.74, 4.09 ± 0.85, and 6.58(±3.82), respectively. Based on the clinical cutoff score of 5, 65.0% of participants were classified as having poor sleep quality. Moreover, correlation analysis showed significant correlations between alcohol use and PTSD symptom severity (r = 0.363, *p* < 0.001), coping motives for drinking (r = 0.615, *p* < 0.001), family support (r = −0.157, *p* < 0.001), and sleep quality (r = 0.319, *p* < 0.001), as presented in Table 3.

### 3.3. Mediating Effect of Sleep Quality and Coping Motives for Drinking in the Relationship Between PTSD Symptom Severity and Alcohol Use

Analyses were conducted using PROCESS Macro Model 85, with age and sex included as covariates. Continuous predictors were mean-centered prior to creating interaction terms. Variance inflation factor (VIF) values ranged from 1.13 to 1.71, indicating no concerns regarding multicollinearity. The mediating effect of sleep quality in the association between PTSD symptom severity and alcohol use was 0.019 (SE = 0.018; 95% BCa CI: −0.017 to 0.054), which included zero and was therefore not statistically significant. In contrast, the indirect effect via coping motives for drinking was 0.086 (SE = 0.022; 95% BCa CI: 0.039 to 0.124), which did not include zero and was statistically significant. Unstandardized coefficients (B), standardized coefficients (β), standard errors (SE), t-values, and 95% confidence intervals (CIs) for each path are presented in Table 4 and Table 5.

**Figure 2:**
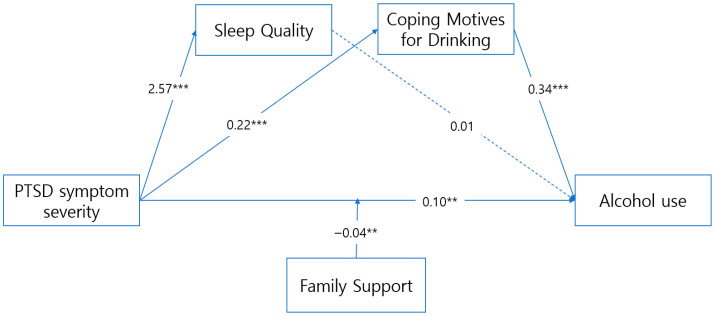
Effects of sleep quality and coping motives for drinking, and the moderating effect of family support on alcohol use. ** *p* < 0.01; *** *p* < 0.001.

These mediating pathways are visually summarized in Figure 2, which illustrates the overall model.

### 3.4. Moderating Effect of Family Support in the Relationship Between PTSD Symptom Severity and Alcohol Use

**Figure 3:**
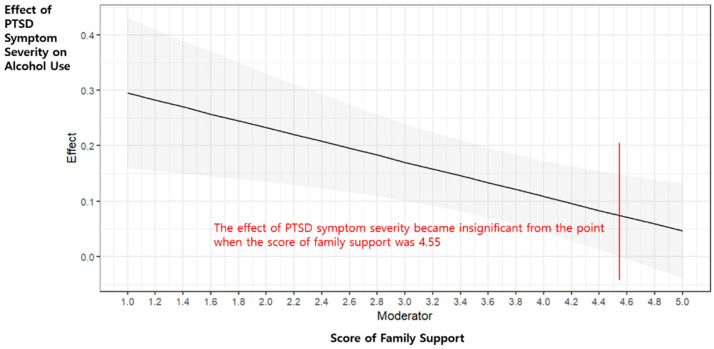
Moderating effect of family support on the relationship between PTSD symptom severity and alcohol use. The shaded area represents the 95% confidence interval.

Family support was found to be a statistically significant moderator in the relationship between PTSD symptom severity and alcohol use (B = −0.04, *p* = 0.007). The Johnson–Neyman technique indicated that the PTSD × family support interaction became non-significant only when family support exceeded 4.55. Given that the mean level of family support in this sample was already high (M = 4.09, SD = 0.85), this threshold is interpreted not as the midpoint of the scale but rather as an empirical cut-off reflecting ceiling effects and range restriction. The conditional effects are depicted in Figure 3.

## 4. Discussion

As firefighters’ issues with alcohol misuse are closely related to the nature of the profession, it is necessary to systematically analyze the interactions between the various factors that affect the issue. This study examined the relationship between PTSD symptom severity, alcohol use, coping motives for drinking, sleep quality, and family support among Korean firefighters to provide foundational data for developing a firefighter-customized intervention program to reduce alcohol misuse.

Statistical analysis showed that PTSD symptom severity was significantly associated with alcohol use and that coping motives for drinking mediated the relationship between PTSD symptom severity and alcohol use. However, sleep quality did not mediate this association, and family support was identified as a significant moderator between PTSD symptom severity and alcohol consumption.

First, PTSD symptom severity was significantly associated with alcohol use among firefighters. Similarly, previous studies have reported that repeated exposure to traumatic events while on duty is highly likely to be associated with alcohol misuse [10,36,37]. PTSD symptoms are characterized by psychological distress, apprehension, and hypervigilance, which may be related to an increased likelihood of alcohol consumption as a short-term avoidance strategy. Although alcohol use can temporarily alleviate PTSD symptoms, long-term alcohol use is associated with a high risk of alcohol dependence [1]. Moreover, reliance on alcohol as a means of coping with PTSD symptoms has been linked to greater difficulty in addressing underlying psychological problems, potentially contributing to a cycle in which PTSD symptom persistence and severity are reinforced over time [18,38]. The strong association between PTSD symptoms and alcohol use indicates that managing PTSD symptoms may be important for resolving firefighters’ alcohol misuse problems.

Second, the mediating effect of coping motives for drinking on the relationship between PTSD symptom severity and alcohol use suggests that firefighters may be more likely to consume alcohol as a means of alleviating stress and negative emotions stemming from PTSD symptoms. According to Cox and Klinger’s motivational model of alcohol use [8], alcohol can be used as a primary coping strategy under stress, and heightened PTSD symptoms may further strengthen this motivation [9,39]. Consistent with this, Lebeaut et al. reported a strong association between PTSD symptoms and coping motives for drinking, observing an increased tendency to drink as PTSD symptoms intensified [10]. In the present study, coping motives for drinking statistically mediated the PTSD–alcohol relationship (B = 0.080, 95% CI [0.039, 0.124]); however, the effect size was modest. Therefore, we cautioned against overemphasizing its clinical significance and interpreted coping motives for drinking as one of several possible mechanisms linking PTSD symptoms to alcohol use. On this basis, interventions for firefighters should not only address coping motives for drinking but also incorporate a broader range of psychological factors and healthy stress management strategies beyond alcohol use.

Third, this study found that sleep quality did not mediate the relationship between PTSD symptom severity and alcohol use, which contrasts with previous research reporting that lower sleep quality has been associated with increased alcohol consumption [12,13]. One possible explanation lies in the generally poor sleep observed among participants. The mean PSQI score was 6.58, indicating reduced sleep quality compared with prior studies using the same instrument [40,41,42]. Notably, 65.0% of participants scored above the clinical cutoff of 5 on the PSQI, indicating a high prevalence of clinically significant poor sleep in this sample. Given this already impaired sleep quality, a ceiling effect may have limited further variability, thereby attenuating the mediating role of sleep quality. In addition, several alternative explanations should be considered. Sleep-related distress may overlap with coping motives for drinking, potentially reducing the unique contribution of sleep quality in the tested pathway. Restricted variability in PSQI scores within this occupational sample may have limited statistical sensitivity to detect indirect effects, and a bidirectional relationship is also plausible, whereby alcohol use exacerbates sleep problems rather than sleep quality functioning as an upstream mediator. Indeed, recent longitudinal and clinical studies have demonstrated bidirectional associations between sleep and alcohol use and have consistently reported poorer sleep quality among individuals with higher alcohol consumption or alcohol-related problems [43,44,45]. Furthermore, the chronic sleep disturbances inherent in firefighters’ occupational demands may have weakened the direct association between sleep and alcohol use. These findings suggest that improving sleep quality alone may not be sufficient to prevent PTSD-related alcohol misuse and that an integrated approach addressing psychological and behavioral factors, such as coping motives for drinking, may be warranted.

Fourth, family support was found to moderate the relationship between PTSD symptom severity and alcohol use. Firefighters with stronger family support showed a weaker association between PTSD symptoms and alcohol use, implying that family support is a critical protective factor for maintaining healthy mental conditions in firefighters. This finding is consistent with a study by Ki-Soo et al. that demonstrated that family support relieves PTSD symptoms and is crucial in reducing negative outcomes [16]. Watkins et al. also reported that family support can act as a psychological buffer in stressful situations and emphasized that it can suppress an increase in alcohol use due to PTSD symptoms [17]. However, in this study, the moderating effect of family support was no longer statistically significant once scores exceeded 4.55. This suggests that when family support reaches a sufficiently high level, its protective effect may already be maximized, thereby limiting additional benefits in reducing PTSD-related alcohol use. Given the relatively high mean level of family support in our sample (M = 4.09, SD = 0.85), this pattern may reflect statistical issues such as ceiling effects or range restriction, as well as the cultural and social context of Korean firefighters, for whom family often constitutes the primary source of emotional and practical support [46]. Accordingly, a certain degree of support may function as a “default” condition, with further increases yielding diminishing returns. Future research should consider non-linear modeling approaches to better capture potential ceiling effects or range restrictions in family support.

In summary, the findings of this study have highlighted important associations relevant to the development of intervention programs for PTSD symptom management and resolving problems related to alcohol use among firefighters. Particularly, a program that strengthens family support may be effective in preventing alcohol misuse due to PTSD symptoms. Firefighters with strong family support can cope better with psychological stress and are less likely to experience drinking-related problems. Therefore, a family-centered mental health support program for firefighters is necessary. Specifically, family education and counseling on PTSD symptoms and alcohol misuse, the creation of family support groups, and the creation of crisis intervention programs could be alternatives [47].

Furthermore, considering the importance of coping motives for drinking, educating firefighters on ways to relieve stress, in addition to alcohol consumption, is a critical intervention strategy. It is essential to develop a program that helps firefighters learn various psychological coping strategies to reduce their coping motives for drinking due to PTSD symptoms. It is necessary to help firefighters find healthy ways to relieve stress via stress management programs using various techniques, such as cognitive behavioral therapy, stress awareness and relaxation, breathing exercises, physical activity, and training on controlling negative emotions [48,49]. Moreover, encouraging firefighters to have peer support can be an effective coping mechanism that can replace maladaptive coping strategies such as drinking alcohol [50]. According to previous studies, peer support programs increase the ability of firefighters with PTSD symptoms and alcohol use disorder to recover and cope with stress and result in better long-term health outcomes by increasing their participation in the treatment [51,52].

## 5. Limitation

The limitations of this study are as follows.

First, this study employed a cross-sectional design in which all variables were measured at a single time point; therefore, the indirect effects estimated using the PROCESS macro with bootstrapping should be interpreted as statistical associations rather than causal mediation effects, because temporal precedence could not be established and reverse causality cannot be ruled out. Second, participants were recruited via a 72 h intranet posting in one metropolitan fire department (City B), yielding a non-probability, self-selected sample. As such, selection bias is possible, and the findings may not generalize beyond similar settings or to other regions or countries. In addition, although most participants were men, the proportion of women in the sample was higher than in the City B firefighter workforce (12.2% vs. 8.1% [32]), which may further limit generalizability. Moreover, the rank distribution of the study sample differed from that of the City B firefighter workforce, with middle-ranking positions being overrepresented, suggesting that differential participation by rank cannot be ruled out. Third, the short data collection period may have been influenced by time-specific factors such as shift schedules and seasonal call patterns, potentially affecting both participation and self-reported outcomes.

In addition, firefighters working within the same crew or fire station are likely to share work schedules, exposure patterns, and organizational climates, which can induce clustering in psychosocial and behavioral outcomes. Because we did not collect identifiers for specific crews or stations, we were unable to account for this potential clustering using multilevel or cluster-robust methods, and the standard errors of individual-level associations may therefore be somewhat underestimated. This should be considered when interpreting the precision and statistical significance of our estimates.

In this study, sleep quality was assessed without distinguishing between sleep obtained during work shifts and sleep obtained at home. Because sleep in these settings may reflect qualitatively different processes among firefighters, this approach may have obscured setting-specific aspects of sleep quality, which should be considered when interpreting the findings. Finally, because eligibility was restricted to firefighters with lifetime alcohol use, the findings of this study have limited generalizability and should be interpreted as applicable mainly to firefighters with drinking experience. This restriction represents a deviation from studies that recruit all firefighters regardless of alcohol use and may limit comparability with research based on fully population-representative firefighter samples.

## 6. Conclusions

The present study explains the complex relationship between PTSD symptom severity and alcohol use among firefighters and provides insights into important variables that can alleviate this relationship. Family support and coping motives for drinking have especially significant implications for maintaining a healthy mental state and preventing alcohol misuse among firefighters. A diversified intervention for firefighters should be developed based on the findings of this study, which will contribute to the development of a healthier coping strategy that effectively relieves the high levels of stress experienced by firefighters on duty. Moreover, future research may further examine whether these associations differ by demographic characteristics such as gender and age, thereby helping to refine more targeted interventions for diverse firefighter populations.

## Figures and Tables

**Table 1:** Demographic and work-related characteristics of the participants (N = 600).

Variable	Item	n	%	Mean ± SD
Gender	Male	527	87.83	
	Female	73	12.17	
Age	20s	88	14.67	38.64 ± 8.57
	30s	263	43.83	
	40s	156	26.00	
	50s	93	15.50	
Marital status	Single	189	31.50	
	Married	402	67.00	
	Other	9	1.50	
Ranks	Firefighter	175	29.17	
	Senior firefighter	103	17.17	
	Fire sergeant	153	25.50	
	Fire lieutenant	133	22.16	
	Fire captain and higher ranks	36	6.00	
Responsibility	Extinguishing fires	166	27.67	
	Firefighter driver/engineer	92	15.33	
	Rescue	23	3.83	
	EMS	161	26.83	
	Operations center	2	0.33	
	Fire investigation	17	2.83	
	Administration	133	22.18	
	Other	6	1.00	
Working years	<5 years	216	36.00	10.76 ± 8.70
	5≤ and <10 years	78	13.00	
	10≤ and <15 years	130	21.67	
	15≤ and <20 years	67	11.17	
	≥20 years	109	18.16	
Service duration	<5 years	274	45.67	8.32 ± 7.60
	5≤ and <10 years	106	17.67	
	10≤ and <15 years	96	16.00	
	15≤ and <20 years	61	10.16	
	≥20 years	63	10.50	
Number of	0 times/week	151	25.17	11.65 ± 13.50
emergency	1–5 times/week	142	23.66	
dispatches	6–10 times/week	91	15.17	
(per week)	≥11 times/week	216	36.00	
Number of	0 nights/month	154	25.67	7.35 ± 5.90
night shifts	1–5 nights/month	90	15.00	
(per month)	6–10 nights/month	177	29.50	
	≥11 nights/month	179	29.83	
Number of drinks containing caffeine	None	53	8.83	
	One cup per day	243	40.50	
	Two cups per day	176	29.33	
	Three cups per day	96	16.00	
	Four cups or more per day	32	5.34	

**Table 2:** Descriptive statistics of the variables.

Variable	Mean ± SD	Max	Total Mean ± SD	Range
PTSD symptom severity (PCL-5) ^1^	1.66 ± 0.76	5.00	33.20 ± 15.20	0–80
Alcohol use (AUDIT-K) ^2^	0.68 ± 0.62	4.00	6.80 ± 6.20	0–40
Coping motives for drinking (Shin & Han)	2.31 ± 0.74	5.00	9.20 ± 3.00	4–20
Family support (Social Support Scale)	4.09 ± 0.85	5.00	102.30 ± 21.30	25–125
Sleep quality (PSQI-K)	6.58(±3.82)	21.00	6.58 ± 3.82	0–21

^1^ PCL-5 probable PTSD ≥ 33; ^2^ AUDIT-K risky drinking ≥ 8.

**Table 3:** Correlations of the variables.

	PTSD Symptom Severity	Alcohol Use	Coping Motives for Drinking	Social Support	Sleep Quality
PTSD symptom severity	1				
Alcohol use	0.363 **	1			
Coping motives for drinking	0.318 **	0.615 **	1		
Family support	−0.319 **	−0.157 **	−0.031 (0.451)	1	
Sleep quality	0.153 **	0.319 **	0.335 **	−0.214 **	1

** *p* < 0.01.

**Table 4:** Regression analysis testing the mediating and moderating effects of the variables on alcohol use.

Variable	B	SE	*β*	t	*p*
PTSD symptom severity	0.10	0.03	0.13	3.08	0.002
Sleep quality	0.01	0.01	0.04	0.92	0.360
Coping motives for drinking	0.34	0.02	0.53	15.15	<0.001
Family support	−0.03	0.02	−0.04	−1.11	0.266
Interaction (PTSD × Family support)	−0.04	0.01	−0.09	−2.73	0.007
R^2^	0.399				
F (*p*)	57.76 (<0.001)				

**Table 5:** Mediating effect of sleep quality and coping motives for drinking on alcohol use.

Route	Effect	Bootstrap SE	95% CI
PTSD symptom severity > Sleep quality >Alcohol use	0.018 (*β* = 0.023)	0.018	−0.017, 0.054
PTSD symptom severity> Coping motives for drinking >Alcohol use	0.080 (*β* = 0.099)	0.022	0.039, 0.124

## Data Availability

The datasets generated and/or analyzed during the current study are not publicly available due to ethical and institutional restrictions, but are available from the corresponding author upon reasonable request.

## Supplementary Materials

The following supporting information can be downloaded at: https://www.mdpi.com/article/10.3390/healthcare14040421/s1, Table S1. Comparison of demographic characteristics between the City B firefighter workforce and the study sample (2021). Table S2. Sensitivity analyses of path coefficients by gender and age.

## Abbreviations

The following abbreviations are used in this manuscript:

PTSD	Post-Traumatic Stress Disorder
PCL-5	PTSD Checklist for DSM-5
PSQI	Pittsburgh Sleep Quality Index
PSQI-K	Korean version of the Pittsburgh Sleep Quality Index
AUDIT	Alcohol Use Disorders Identification Test
AUDIT-K	Korean version of the Alcohol Use Disorders Identification Test
