# Supplementary material for: Posttraumatic Stress Disorder Symptoms and Alcohol Use Among Korean Firefighters: The Roles of Coping Motives for Drinking and Family Support
Source: Healthcare (Basel). 2026 Feb 7;14(4):421. doi: 10.3390/healthcare14040421 (PMC12940857; doi:10.3390/healthcare14040421)
Supplement: Supplementary file 1 [file healthcare-14-00421-s001.zip › healthcare-4095808-supplementary.pdf]

Supplementary Table S1. Comparison of demographic characteristics between the City B firefighter workforce and the study sample (2021)*

Variable		City B Firefighter Workforce (%)	Study Sample (N = 600) (%)
Gender			
	Male	91.88	87.83
	Female	8.12	12.17
Rank			
	Firefighter	43.71	29.17
	Senior firefighter	25.01	17.17
	Fire sergeant	13.14	25.50
	Fire lieutenant	8.10	22.16
	Fire captain and higher ranks	10.05	6.00

*Population-level demographic data for City B firefighters were obtained from the National Fire Agency Statistical Yearbook (2021). Age distribution data for the City B firefighter workforce were not available and are therefore not included.

Supplementary Table S2. Sensitivity analyses of path coefficients by gender and age

Pathway	Male (B, SE)	Female (B, SE)	Age 20s-30s (B,SE)	Age 40s-50s (B,SE)
PTSD → Sleep quality	2.72 (0.18)	2.67 (0.51)	2.67 (0.23)	2.77 (0.27)
Sleep quality → Alcohol use	0.05 (0.01)	0.06 (0.02)	0.04 (0.01)	0.07 (0.01)
PTSD → Coping motives	0.43 (0.05)	0.35 (0.13)	0.44 (0.07)	0.36 (0.07)
Coping motives → Alcohol use	0.38 (0.02)	0.39 (0.06)	0.36 (0.03)	0.42 (0.04)

Note. B = unstandardized regression coefficient; SE = standard error. Sensitivity analyses were conducted using the same analytic models as the primary analyses. Age groups were created by collapsing decade-based categories into 20s–30s and 40s–50s to ensure adequate subgroup sizes and consistency with Table 1. Although estimates among female firefighters were less precise due to the smaller subgroup size, the direction of associations was consistent across all subgroups. All coefficients were statistically significant ($p < .05$).
